# A carbonised sieve-like corn straw cellulose–graphene oxide composite for organophosphorus pesticide removal†

**DOI:** 10.1039/c7ra12898c

**Published:** 2018-02-16

**Authors:** Fengyue Suo, Guixian Xie, Jie Zhang, Jingyu Li, Changsheng Li, Xue Liu, Yunpeng Zhang, Yongqiang Ma, MingShan Ji

**Affiliations:** College of Plant Protection, Shenyang Agricultural University Shenyang 110866 China; College of Resources and Environment, Hunan Agricultural University Changsha 410128 China; College of Science, China Agricultural University Beijing 100193 China

## Abstract

The development of efficient adsorbents for the removal of organophosphorus pesticides from water is a major challenge. In this work, we prepared an activated carbon derived from sieve-like cellulose/graphene oxide composites (ACCE/G) for the removal of several organophosphorus pesticides. We employed corn straw to produce a sieve-like cellulose–graphene oxide composite (CCE/G); then, by treating CCE/G with potassium hydroxide at high temperatures, the efficient adsorbent ACCE/G was prepared. The adsorption capacity of ACCE/G is higher than those of other sorbents, including a multi-wall carbon nanotube, graphitised carbon black, activated carbon, C18, and primary secondary amine adsorbent. The ACCE/G structure has been fully characterised *via* scanning electron microscopy, Fourier-transform infrared spectroscopy, X-ray photoelectron spectroscopy, X-ray diffraction and Brunauer–Emmett–Teller analysis. The maximum adsorption capacity of ACCE/G is 152.5 mg g^−1^ for chlorpyrifos. The mechanism, the thermodynamic properties, and the kinetics of the adsorption process have been investigated as well. Our findings demonstrate that the adsorption mechanism depends on the electron-donating abilities of the S and P atoms. Moreover, the Langmuir model gives the best fit for the isotherm data, and the adsorption efficiency of the ACCE/G is still over 80% after eight times of recycling, making ACCE/G a valuable candidate for the removal of OPPs.

## Abstract 2

Synthesizing a reusable adsorbent from waste corn straw is a sustainable way to utilize secondary resources and purify water.

## Graphical abstract

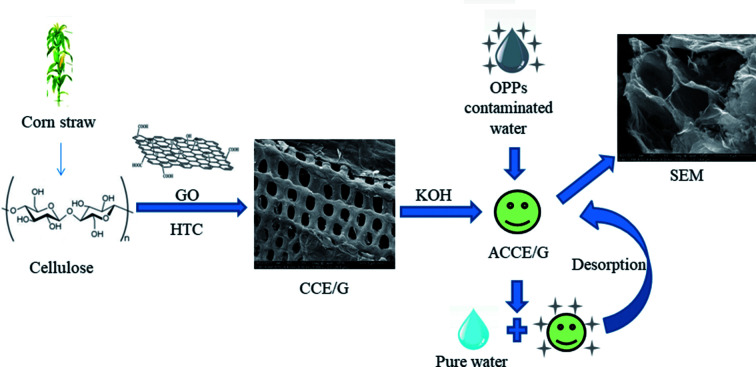

## 1. Introduction

Organophosphorus pesticides (OPPs) are one of the most extensively used compounds to protect crops.^1^ However, the abuse of highly-toxic compounds leads to the pollution of the environment and of the crop and poultry products, eventually causing food safety issues.^2^ Several methods have emerged as promising techniques for the removal of OPPs, such as advanced oxidation, enzymatic biodegradation, and adsorption techniques.^3–6^ Compared to the other methods mentioned above, adsorption has the advantages of low cost, easy to operate, and wide range of applications. Therefore, it is important to develop an efficient adsorbent for the removal of OPPs.

Corn straw is the typical source of cellulose because of its large-scale output across the world.^7^ The annual production of corn straw is ∼350 million tons in China. In corn straw, the content of cellulose is in the range of 28–36%. Nowadays, most of the corn straw has been either employed to improve the soil fertility or burnt contributing to pollution.^8^ The cellulose, derived from natural sources, shows adsorption properties in various forms, including raw cellulose, modified cellulose, and as a precursor to activated carbon (AC). These distinct forms of cellulose can be used to remove pollutants, such as dyes, heavy metals, pesticides, and antibiotics.^9–12^ Recently, the use of AC derived from cellulose has been widely explored because of its high adsorption properties. Laszlo *et al.* prepared porous carbon utilising packing material as a source of cellulose to remove phenol from aqueous solution.^13^ However, further systematic effort on the development of methods to efficiently derive AC from cellulose precursors is required. Moreover, the adsorption mechanisms for the removal of pollutants from wastewater also need to be investigated in more detail. Indeed, few studies have been published to explain the different adsorption mechanisms, such as the high surface area^14^ and the effect of functional groups within adsorbents. Huang *et al.* synthesised an adsorbent, based on a modified ligno-cellulosic material, which can remove cadmium and lead from aqueous solutions. They found that the sorption was a chemical process involving surface chelation and ion exchange.^15^

Graphene oxide (GO), the precursor of graphene, is a two-dimensional (2D) carbon sheet with oxygen-containing functional groups.^16^ Due to its conductivity, GO can easily be combined with polymers and other materials, forming composite materials with enhanced properties, such tensile strength, elasticity, and conductivity.^17^ According to previous studies, GO and its derivatives can also work as absorbents for the removal of antibiotics, metal ions, dyes, and pesticides.^18–21^

Herein, we report on the synthesis of AC which is derived from a novel sieve-like cellulose–graphene oxide composite. The cellulose was extracted from corn straw which is biodegradable, biocompatible, and economical. The so-synthesised composite material was successfully used as an adsorbent to remove OPPs from contaminated water. The physical–chemical characteristics of the so-prepared composite had been assessed as well. Finally, the adsorption performance and the mechanisms for the removal of OPPs from the water were also discussed systematically.

## 2. Experimental methods and materials

### 2.1 Materials

Corn straw was supplied by a local farm of Northeast China. After cut into small pieces (length 2–3 cm), the corn straw was washed with deionised water and then dried at 60 °C for 24 h. The dried material was then ground into powder. Dichlorvos, dimethoate, chlorpyrifos, chlorfenvinphos, methidathion, and profenofos were obtained from the Institute of the Control of Agrochemicals, Ministry of Agriculture, China. Chromatographic grade ethyl acetate was purchased from Mreda Co., Ltd. (Beijing, China). Graphite was obtained from Qingdao Hensen Graphite Co., Ltd. (Qingdao, China). Activated carbon (surface area/1000 m^2^ g^−1^) was obtained by Sinopharm Chemical Reagent Co., Ltd. (Shanghai, China), whereas primary secondary amine adsorbent (PSA, surface area/480 m^2^ g^−1^), graphitised carbon black (GCB, surface area/200 m^2^ g^−1^), and C18 (surface area/600 m^2^ g^−1^) were obtained from Bonna-Agela China (Tianjin, China).

### 2.2 Preparation of the graphene oxide

GO powder used in this work was obtained according to modified Hummers' method.^22^ Detailed experimental steps can be referred to our previous works.^23^

### 2.3 Preparation of the cellulose from corn straw

Cellulose was extracted from corn straw with modified Kurschner–Hoffer method.^24^ 20 g of corn straw powder was treated with 500 mL HNO_3_ : EtOH mixture (1 : 4, v/v) under reflux and stirred at 100 °C until the powder was turned into white. The product was washed with deionised water and then was dried at 60 °C for 12 h.

### 2.4 Synthesis of the AC derived from cellulose–graphene oxide composite (ACCE/G)

According to the method used by Zheng *et al.* for the preparation of a porous graphene/activated carbon composite,^25^ 0.05 g of GO powder was first added to 50 mL of deionised water and then dispersed by ultrasonication with a power of 100 W for 30 min. After that, 1.5 g of cellulose and 2.0 g of urea were added. After ultrasonication for other 30 min, the mixture was transferred into Teflon autoclave and hydrothermally treated at 180 °C for 24 h. After filtering and washing with deionised water and ethanol, the obtained solid sample was dried at 60 °C under vacuum for 12 h to obtain the intermediate product: carbonised cellulose–graphene oxide composite (CCE/G). The CCE/G was mixed with KOH at different weight ratios (0, 1/4, 1/2, 4/1) and was heat treated (400 °C, 500 °C, 600 °C) for 1 h, 2 h, 3 h in a crucible introduced into a muffle furnace connected to the outside atmosphere. After washed with 1 mol L^−1^ HCl solution and deionised water, the obtained product was dried at 60 °C for 12 h to obtain the activated carbonised cellulose–graphene oxide composite (ACCE/G). The AC produced from corn stalk cellulose without incorporation of graphene oxide (ACCE) was also prepared.

### 2.5 Characterisation

The samples were characterised *via* scanning electron microscopy (SEM, Quanta FEG 250), transmission electron microscopy (TEM, Tecnai G2 F20), diffuse reflectance infrared Fourier transform spectroscopy (DRIFT, Spectrum 100 FT-IR), and Raman spectroscopy (Renishaw RM2000). The crystallinity of the sample was investigated by X-ray diffraction (XRD, BRUKER D8 ADVANCE), whereas the Brunauer–Emmett–Teller (BET) surface area (Micromeritics ASAP2020), pore volume, and pore size were characterised using nitrogen adsorption at liquid nitrogen temperatures, and *via* X-ray photoelectron spectroscopy (XPS, Thermo SCIENTIFIC ESCALAB 250Xi).

### 2.6 Adsorption experiments

Different ACCE/G doses (5 to 120 mg) were investigated to determine the proper doses for removing the OPPs. To test the effect of time on adsorption, 50 mg ACCE/G was added to the freshly prepared solution of OPPs (10 mL, 2 mg L^−1^). The samples were stirred on a vortex mixer for 0 (shaken five times by hand), 0.5, 1, 1.5, 2, 2.5, 3 and 5 min. The pHs of the solutions containing OPPs (1–11) were also examined (10 mL, 2 mg L^−1^). The kinetic study was carried out in several 10 mL centrifuge tubes containing 5 mL of chlorpyrifos solution (2 mg L^−1^). The centrifuge tubes were sealed and placed in an oscillator at a speed of 170 rpm with a temperature of 298 K for different durations (1 min, 3 min, 5 min, 7 min, 10 min, 20 min, 30 min, 60 min, 120 min, 180 min, 300 min). To obtain the adsorption isotherm, 5 mg ACCE/G was added to the chlorpyrifos solution (5 mL) with a concentration ranging from 1 to 120 mg L^−1^. Then, the centrifuge tubes were sealed and placed in an oscillator at a speed of 170 rpm with a temperature of 298 K, 308 K, and 318 K, until equilibrium was reached.

After the adsorption process, the solution was centrifuged for 5 min at 3800 rpm; the supernatant was discarded. Ethyl acetate (10 mL), used as a desorption solvent, and NaCl (1 g) were added, and the mixture was vortexed for 3 min to desorb the analytes. Then the mixture was centrifuged at 3800 rpm for 5 min. 1 mL of the supernatant was dehydrated by anhydrous MgSO_4_ (50 mg), after vortexed for 1 min and centrifuged at 10 000 rpm for 1 min. The concentrations of the OPPs in the supernatant were determined by the gas chromatography with flame photometric detection (GC-FPD). The temperature of the injector was held at 220 °C in a splitless mode. The oven temperature was programmed as follows: it was initially held for 1 min at 80 °C, then increased to 180 °C at the rate of 25 °C min^−1^, and further increased to 260 °C at the rate of 15 °C min^−1^. The temperature of the detector was set at 260 °C. The injection volume was 1 mL. Ultrapure nitrogen was used as the carrier gas with a constant linear velocity of 14 mL min^−1^. The flow rates of hydrogen and no air were 80 mL min^−1^ and 120 mL min^−1^, respectively.

## 3. Results and discussion

### 3.1 Morphology and structures of CCE/G

**Fig. 1:**
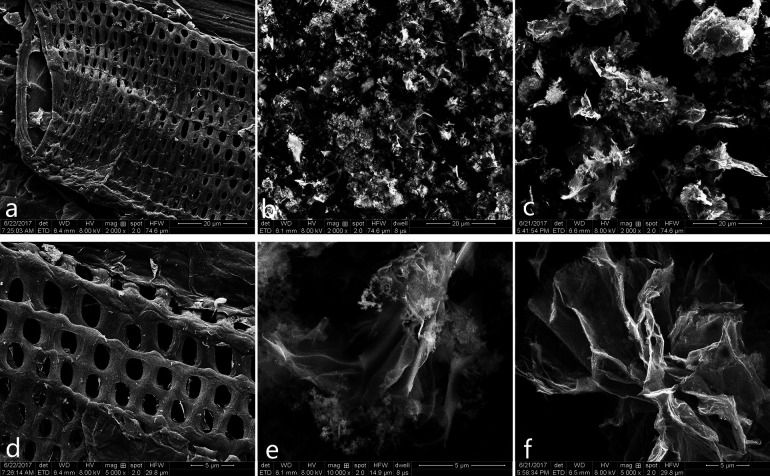
SEM images of CCE/G (a) and (d); SEM images of ACCE (b) and (e); SEM images of ACCE/G (c) and (f).

SEM technique has been utilised to observe the surface morphology of the products. The top view of CCE/G (Fig. 1a) displays a layer with ordered macrospores. Pores can also be observed on the surface of cellulose in Fig. S1a and b.† Munro *et al.* found that nitric acid could cause “surface cracking” on cellulose.^26^ After the dehydration polymerisation in the process of hydrothermal carbonisation, the size of the pores increases, and the sieve-like structure is formed (Fig. S1c†). Cellulose has a larger particle size than GO (Fig. S1b and d†). The surface of CCE (prepared without the presence of GO) is clean and smooth (Fig. S1e†), whereas CCE/G (prepared in the presence of GO) has the sieve-like structure similar to that of CCE. GO nanoparticles are tightly attached to the surface of cellulose (Fig. 1a and d). After activation by KOH, the particle size of ACCE is smaller than that of ACCE/G (Fig. 1b and c), as further confirmed *via* XPS. Both the high-resolution images of ACCE and ACCE/G exhibit a porous structure (Fig. 1e and f). However, the image of ACCE/G shows wrinkled nanosheet morphology. TEM images of ACCE/G (Fig. S1c†) further confirm that the presence of GO promotes the formation of porous nanosheets.

**Fig. 2:**
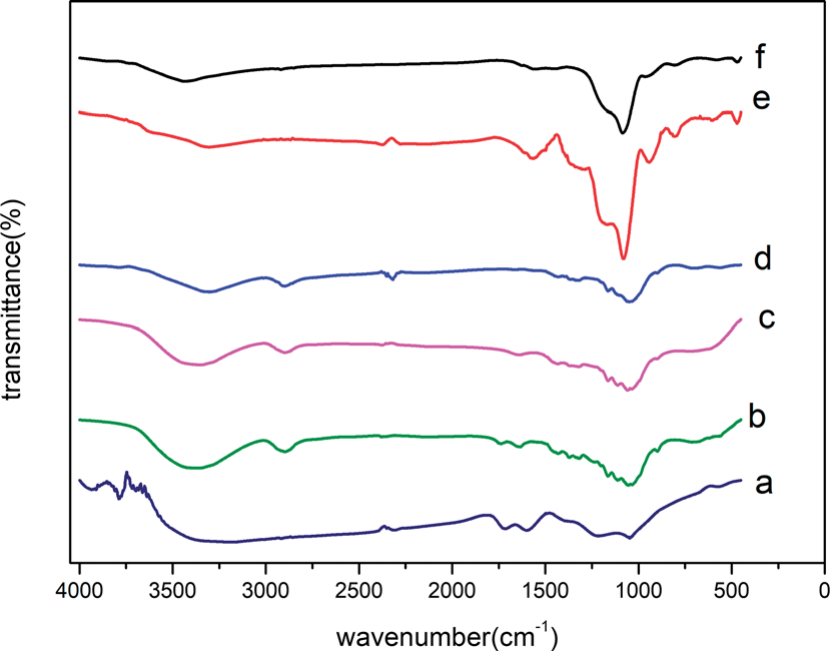
FT-IR spectra of GO (a), cellulose (b), CCE (c), CCE/G (d), ACCE (e), and ACCE/G (f).

We have conducted FT-IR experiments to provide insight into the structures of CCE, CCE/G, ACCE, and ACCE/G. The FT-IR spectra show that the absorbance peaks at 3382 cm^−1^, 2897 cm^−1^, 1370 cm^−1^, 1319 cm^−1^, 1164 cm^−1^, 1112 cm^−1^, 1060 cm^−1^, and 897 cm^−1^ are associated with the cellulose (Fig. 2b). The structures of cellulose, HTC cellulose (CCE) and HTC prepared in the presence of GO (CCE/G) are, indeed, similar (Fig. 2), which can be further confirmed *via* XRD (Fig. S2†). However, the absorption bands of CCE/G are weaker and broader than those of cellulose, suggesting that dehydration and aromatisation reactions take place to a higher extent in this case than during the hydrothermal carbonisation.^14^ The absorbance peaks of OH groups shift from 3382 cm^−1^ to 3261 cm^−1^, whereas the absorbance peak associated to the vibration mode of the C–O–C pyranose-ring skeletal moves from 1060 cm^−1^ for CCE to 1052 cm^−1^ for CCE/G.^27,28^ This phenomenon may indicate the presence of an interaction between the carboxy groups of GO and the oxygen groups in the cellulose. The treatment with KOH modifies the spectra of the composite materials. The peaks of ACCE and ACCE/G are weaker and broader than those of CCE and CCE/G. The heat treatment with KOH leads to a graphitic/aromatic structure with fewer oxygen-containing groups. This phenomenon is further confirmed *via* XPS: the peaks of ACCE/G are weaker than those of ACCE, suggesting that the presence of GO could enhance the level of aromatisation.

X-ray diffraction experiments have been performed to explore the crystallographic structures of the composite materials as well. In the Fig. S2a,† according to the characteristic diffraction peaks of cellulose,^29^ the spectra of CCE and CCE/G exhibit two main peaks at 2*θ* = 16.18° and 22.64°, which correspond to the (110) and (200) planes. This suggests that cellulose, CCE, and CCE/G have the same crystallographic structure. However, the intensity of these two peaks increases slightly passing from CCE to CCE/G due to the presence of GO, which may lead to a higher polymerisation degree.^30^ Because of the volatile reducing gases produced during hydrothermal carbonisation, the presence of urea can restore p-conjugation structure and promote the removal of oxygen-functional groups.^31^ The treatment with KOH has influenced the X-ray diffraction spectra of ACCE and ACCE/G. The peaks of ACCE and ACCE/G become weaker and broader, probably suggesting that a drastic morphological transformation takes place during the activation process^32^ and that the particle size of the composite has been reduced during the chemical activation process.^25^ The peak of ACCE was broader than that of ACCE/G, which indicates that the particle size of ACCE was smaller than ACCE/G.

Raman spectroscopy has been applied to investigate the carbon structures of CCE/G, ACCE, and ACCE/G. Fig. S2b† illustrates the Raman patterns of the CCE/G, ACCE, and ACCE/G. The D and G peaks at 1352 cm^−1^ and 1598 cm^−1^, can be observed for the three composite materials. The intensity of the D band at 1352 cm^−1^ is attributed to the presence of condensed benzene rings and/or defected graphitic structures. Therefore, the presence of the D band in the Raman spectra of the ACCE and ACCE/G confirms the formation of small aromatic clusters during chemical activation. The G band at 1598 cm^−1^ originates from the sp^2^ hybridisation of C–C bonds.^33^ The ratio of the intensity between D band and G band for ACCE and ACCE/G increases from 0.84 to 0.98; this increased defect degree is apparently due to the increased ratio of sp^3^ hybridised carbon in the presence of GO. The broad band at 2680 cm^−1^ of ACCE/G and ACCE can be assigned to the 2D peak, which is an overtone of the D band. The appearance of a well-defined 2D band is known to be related to the development of a significant graphitic character in carbon materials.^16^

**Fig. 3:**
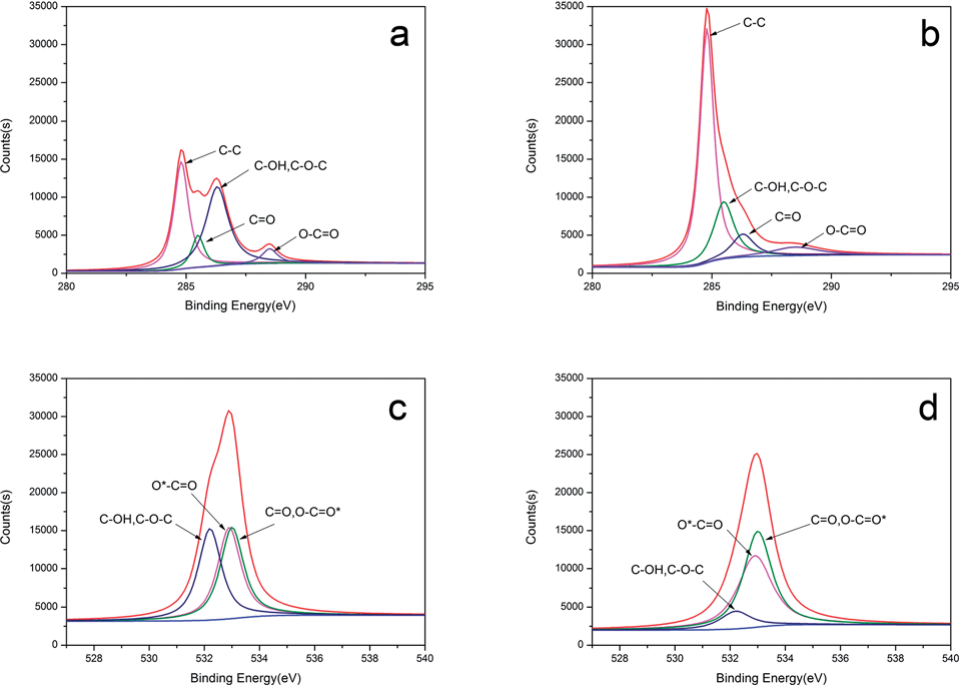
XPS images of CCE/G C 1s (a), ACCE/G C 1s (b), CCE/G O 1s (c), and ACCE/G O 1s (d).

The component elements have been further analysed *via* surface sensitive high-resolution XPS to determine the composition of the materials before and after the KOH treatment. The C 1s binding energies are 284.2 eV, 284.8 eV, 285.5 eV, 286.3 eV, and 288.5 eV (Fig. 3a and b) for the following bonds: C–C, C–OH/C–O–C, C=O, and O=C–O.^34^Fig. 5c and d show the O 1s spectrum can be fitted to three component peaks at 532.9 eV, 532.2 eV, 533.0 eV, which represent C–O/O–C–O* groups, C–OH and/or C–O–C groups, *O–C–O, respectively.^35^ After activation by KOH, the content of carbon increases, while the content of oxygen decrease, which is in agreement with the FT-IR spectra.

**Fig. 4:**
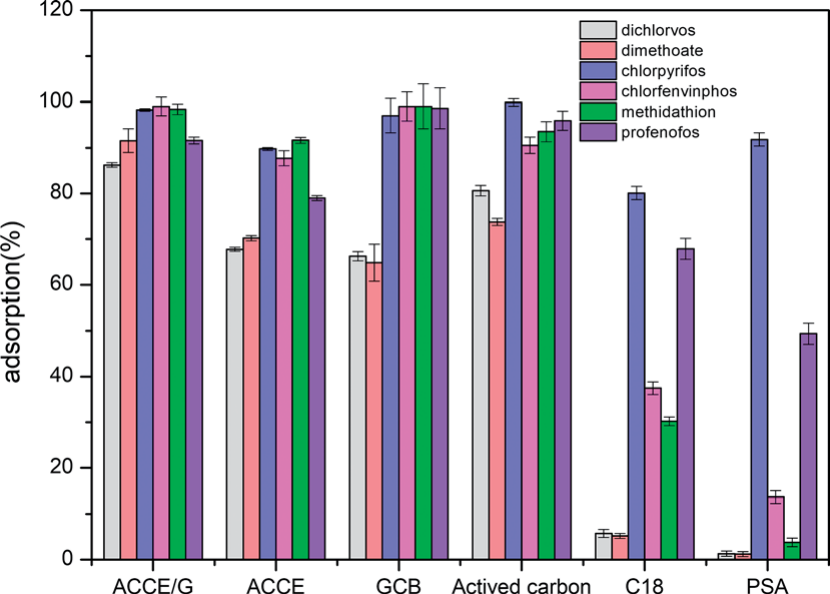
Comparison of the adsorption efficiency of ACCE/G, GCB, AC, C18, PSA, and ACCE towards six OPPs.

**Fig. 5:**
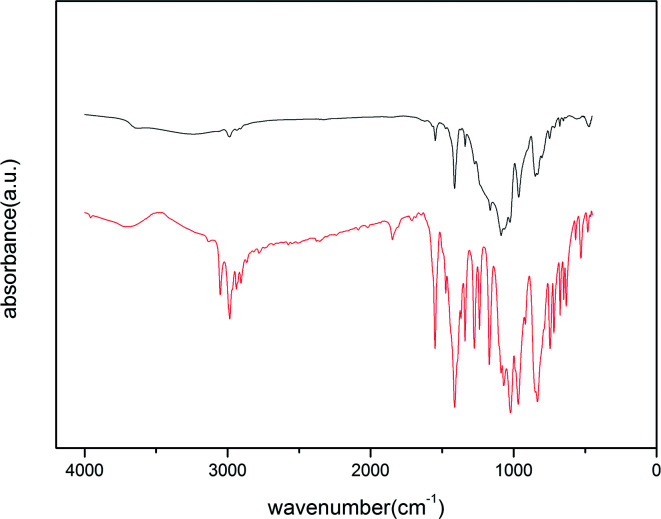
IR spectra of chlorpyrifos (first line) and ACCE/G after adsorption of OPPs in water (second line).

Detailed information on the BET surface area and pore size distribution of the CCE/G and ACCE/G samples are summarised in Table 1. The porosity has substantially increased during the heat treatment with KOH. After the activation process, the BET of ACCE/G dramatically increases to 160 m^2^ g^−1^, which is almost 18 times that of CCE/G (9.0160 m^2^ g^−1^). Therefore, it is evident that the process of activation plays an essential role in the creation of porosity.^36^ The increase of the BET and the volume of microporous may be responsible for the OPPs adsorption.

**Table 1:** Textural parameters calculated from the N_2_ (−196 °C) adsorption isotherms for the materials used in this work

Material	*S* Bet (m^2^ g^−1^)	Volume total (cm^3^ g^−1^)	Diameter average (nm)
CCE/G	9.0137	0.014299	6.33296
ACCE/G	160.3765	0.154315	3.84881

### 3.2 Optimisation of the ACCE/G

After activated by KOH, the adsorption capacity of the ACCE/G has been increased dramatically. The adsorption capacity of ACCE is not excellent towards dichlorvos and dimethoate when compared with that of the ACCE/G. Although the adsorption of the other four OPPs (chlorpyrifos, chlorfenvinphos, methidathion, profenofos) are more than 80%, the ACCE/G has a better adsorption capacity than ACCE (Fig. S3a†).

We have optimised the synthesis condition to obtain the most adsorbing ACCE/G. By modulating the temperature and the time of the HTC, we found that these two parameters have little effect on the adsorption ability of the ACCE/G. Therefore, we have varied the conditions of the process of activation by KOH. After chemical activation (HTC/KOH = 1/4, heat-treated 500 °C for 2 h), the average adsorption efficiency of the composite has been increased dramatically. The best weight ratio is HTC/KOH = 1/4 (Fig. S3b†). To determine the optimal temperature, the CCE/G composite was heat-treated for 2 hours at 400 °C, 500 °C, and 600 °C with KOH (HTC/KOH = 1/4). However, when the temperature has reached 600 °C, there is a little amount of product left. As shown in Fig. S3c,† the adsorption capacity of the composite is higher when the heat-treating temperature is 500 °C. After heat-treated for 2 hours and 3 hours, no significant difference can be observed in the adsorption capacity of the product (Fig. S3d†). For economic and environmental concerns, we have chosen the heat-treatment of 2 hours as the standard treatment in this experiment.

### 3.3 Factors affecting the adsorption performance

In this study, the absorbent dose, the adsorption time, the desorption time, and the pH of the solution have been optimised to achieve maximum adsorption efficiency.

#### 3.3.1 Effects of dosage on adsorptive capacity of ACCE/G

The dose of adsorbent plays a crucial role in the adsorption efficiency. Different ACCE/G doses in the range of 5–150 mg (5 mg, 10 mg, 20 mg, 30 mg, 50 mg, 70 mg, 100 mg, 120 mg, and 150 mg) have added to 10 mL solutions containing 2 mg L^−1^ of each of the six organophosphorus pesticides. We have observed that the adsorption capacity has increased with increasing the adsorbent dosage because more surface area is available for adsorption (Fig. S4a†). However, the data of 50 mg shows equally good adsorption performance as 70 mg and 100 mg. Hence, 50 mg of adsorbent was chosen as the standard treatment in this experiment for economic concerns.

#### 3.3.2 Effects of time on adsorptive capacity of ACCE/G

The time and method of mixing are key factors affecting the adsorption efficiency as well. In this test, 50 mg of ACCE/G has been added to the solution and mixed by vortexing for different durations (0 min, 0.5 min, 1 min, 1.5 min, 2 min, 2.5 min, 3 min, and 5 min) before the solutions have been tested. Fig. S4b† illustrates that no stirring (*i.e.*, 0 min, the solution was just shaken five times by hand) produces almost the same effect as stirring for 2.5 min, with the exception of dichlorvos and dimethoate. When the time of vortexing reaches 2.5 min, the adsorption of all the six OPPs exceeds 90%. Therefore, vortexing for 2.5 min was chosen as the standard treatment in this experiment.

#### 3.3.3 Effects of pH on adsorptive capacity of ACCE/G

For water samples, pH is a key factor affecting the ionic states of the target analytes and may, therefore, influence the adsorption properties of the ACCE/G. The pH of the solution was adjusted to 1, 3, 5, 7, 9, and 11 with HCl or NaOH. The data in Fig. S4c† shows no significant variation in adsorption over the pH range studied (pH = 1–7). Hence, no pH adjustments were applied in the following tests.

### 3.4 Comparison with six other sorbents

Six sorbents, including ACCE/G, multi-wall carbon nanotube, GCB, AC, C18, PSA, ACCE, have been tested, and their adsorption performance has been compared. The tests have been performed under the optimal conditions. Fig. 4 presents the results, showing that the adsorptive capacities of the PSA and C18 are unsatisfactory. The adsorption capacity of GCB, AC, and ACCE are not excellent for the cases of dichlorvos and dimethoate. Overall, the ACCE/G shows more performing adsorption capacity than the other five sorbents.

### 3.5 Adsorption mechanism of ACCE/G for six OPPs

The adsorption mechanism involving OPPs is complex since the OPPs contains several functional groups that would interact with the sorbent. According to our findings, the ACCE/G has a high surface area with a considerable amount of mesopores. Moreover, the strong π-bonding network in the OPPs and the electron-donating abilities of the N, S, and O atoms can also generally assist adsorption. In addition, the changes of ACCE/G after residual adsorption in IR (Fig. 5) shows that after the adsorption of chlorpyrifos, the P=S peak has moved from 834 cm^−1^ to 845 cm^−1^, and the P–O–C of aromatic has shifted from 1701 cm^−1^ to 1163 cm^−1^, indicating that the P, S, and O atoms have promoted the adsorption. Although a study suggested that the adsorption mechanism was based on the strong π-bonding network of benzene rings,^23^ the band corresponding to the pyridine-ring vibration has not been modified after the adsorption. The possible reason may be that the pyridine ring is an electron-deficient group; further, the atom C1 attached to the pyridine is an electron withdrawing group. This may occur due to the weakened π-bonding network of the pyridine ring. In summary, the electron-donating abilities of P and S atoms promote the adsorption.

#### 3.5.1 Adsorption kinetics

The adsorption rate and adsorption equilibrium time play a key role in the adsorption mechanism. To further understand the kinetics of the adsorption process, the experimental kinetic adsorption data of chlorpyrifos have been introduced into two kinetic models: the pseudo-first-order model and the pseudo-second-order model. The Lagergren pseudo-first-order model is based on the assumption that the adsorption process has a rapid initial phase.^37^ The model is expressed by the following equation:1
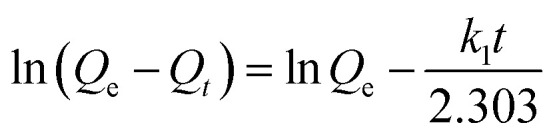
where *Q*_e_ and *Q*_*t*_ are the amounts of chlorpyrifos (mg g^−1^) adsorbed on adsorbent at equilibrium and at a given time *t* (min), whereas *k*_1_ is the rate constant of adsorption (g mg^−1^ min^−1^).

The experimental values *Q*_e_ are not in agreement with the calculated ones as obtained from the linear plots. Moreover, the values of the correlation coefficient (*R*^2^) are relatively low (Fig. S5a†), indicating that the adsorption process does not comply with the pseudo-first-order rate model.

The pseudo-second-order rate model is based on the assumption that the adsorption is rate-controlling which can be expressed by the following equation:^38^2
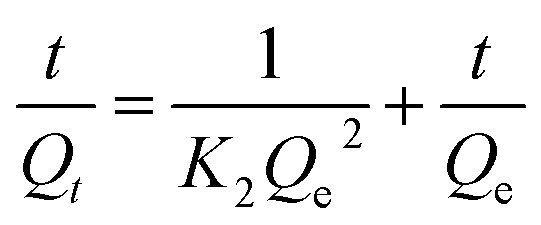
where *K*_2_ is the rate constant for the pseudo-second-order adsorption process.

The linear plots of *t*/*Q*_*t*_*versus t* show a correlation between experimental and calculated values at different initial concentrations (Fig. S5b†). The *R*^2^ value for the pseudo-second-order kinetic model (Fig. S6†) is much higher than the value associated to the pseudo-first-order kinetics model, suggesting that the experimental data fit the pseudo-second-order kinetic model (*R*^2^ > 0.99).^39^ Similar results were acquired by He *et al.*^37^ and Cheng *et al.*^40^ They used treated biofilms as biosorbents for the heavy metals removal.

#### 3.5.2 Adsorption isotherms

In this paper, chlorpyrifos has been selected to derive the adsorption isotherms of ACCE/G. Fig. S5c† shows the adsorption isotherms of chlorpyrifos on ACCE/G over the concentration from 1 to 100 mg L^−1^ at 298 K, 308 K, and 318 K. To understand the chlorpyrifos adsorption behaviours on the ACCE/G, two equilibrium models have been applied to evaluate the experimental data. The Langmuir model is based on the hypothesis that the absorption of metal ions occurs on a homogeneous surface through a single layer. However, the Freundlich model is founded on the adsorption on the heterogeneous surface reference. The two models can be described using eqn (3) and (4), respectively:^41^3
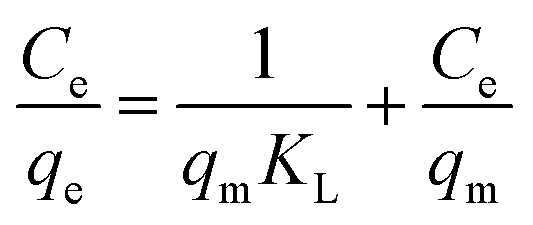
4
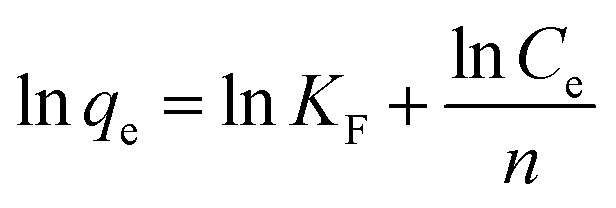
where *K*_L_ (L mg^−1^) is the Langmuir equilibrium constant, *q*_m_ is the maximum adsorption capacity (mg g^−1^), and *K*_F_ ((mg g^−1^) (L mg^−1^)^1/*n*^) and *n* are the Freundlich constants. The Freundlich constants are related to the adsorption capacity and adsorption intensity: with the increase the *K*_F_ and *n* values, the adsorption capacity of adsorbent rises as well.^42^

For Langmuir isotherm model, the correlation coefficients *R*^2^ are larger than 0.96, which are higher than those for the Freundlich model (Table 2). The suitable fit of Langmuir model indicates that chlorpyrifos likely adsorbs forming monolayer coverage on the surface of ACCE/G.^43^ Dried activated sludge was used by Yang *et al.* as a biosorbent for zinc(ii) removal, and also found that the adsorption takes place on a homogeneous surface by monolayer sorption as well.^41^ Furthermore, the maximum adsorption capacities and *K*_L_ for chlorpyrifos on the ACCE/G increases with increasing temperature, indicating that higher temperature is more favorable to the adsorption process.

**Table 2:** Langmuir and Freundlich constants and correlation coefficients (*R*^2^) for chlorpyrifos adsorption on the ACCE/G

*T* (K)	Langmuir			Freundlich		
	*Q*_m_ (mg g^−1^)	*K*_L_ (L mg^−1^)	*R* ^2^	*n*	*K*_F_	*R* ^2^
298	120.48	0.8218	0.9842	1.2015	60.21	0.9768
308	135.14	1.6817	0.9976	1.4753	96.45	0.9799
318	152.52	5.9998	0.9697	2.6137	97.56	0.7983

**Table 3:** Thermodynamic parameters for the adsorption of chlorpyrifos on ACCE/G

*T* (K)	*K*_0_	Δ*G*_0_ (kJ mol^−1^)	Δ*H*_0_ (kJ mol^−1^)	Δ*S*_0_ (J mol^−1^ K^−1^)
298	4.6801	−3.8237	19.7657	78.9946
308	5.7083	−4.4606		
318	7.7399	−5.4104		

#### 3.5.3 Effect of the temperature on the adsorption

We have also conducted experiments 298 K, 308 K, and 318 K to study the thermodynamics of the adsorption process of chlorpyrifos. The thermodynamic parameters can be calculated by the Van't Hoff equation:^44^

5 Δ*G*^0^ = −*RT* ln *K*_0_

6
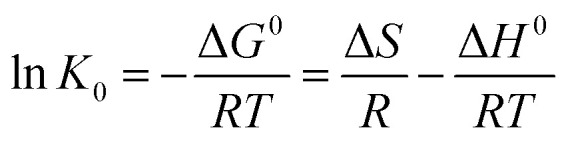


In eqn (5) and (6), *R* is the gas constant (8.314 J mol^−1^ K^−1^); *T* is the sorption temperature in Kelvin; *K*_0_ is the thermodynamic equilibrium constant, which is defined as follow:7
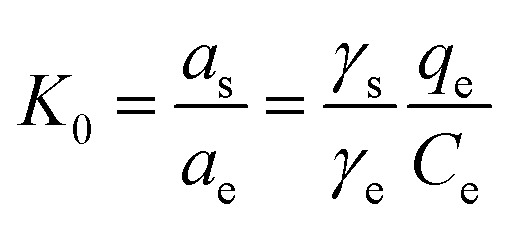


Here, *a*_s_ is the activity of the adsorbed chlorpyrifos, *a*_e_ is the activity of the chlorpyrifos in the solution at equilibrium, *γ*_s_ is the activity coefficient of the adsorbed chlorpyrifos, and *γ*_e_ is the activity coefficient of chlorpyrifos in solution at equilibrium.

As the chlorpyrifos concentration in the solution decreases and approaches zero, the values of *K*_0_ can be acquired by plotting ln(*q*_e_/*C*_e_) *versus q*_e_ and by extrapolating *q*_e_ to zero. Moreover, Δ*G*_0_ can be calculated using eqn (5); Δ*H*_0_ and Δ*S*_0_ can be obtained as the slope and the intercept of the eqn (6).^39^

Gibbs free energy values of the adsorption of chlorpyrifos on ACCE/G decrease with increasing temperature, indicating that the adsorption is a spontaneous and feasible process. The change of free energy for physical adsorption is in the range of −20 to 0 kJ mol^−1^, while chemical adsorption ranges from −400 to −80 kJ mol^−1^.^45^ Furthermore, the processes are endothermic since the enthalpy free energies values are. The positive value of Δ*S*_0_ indicates an increasing disorder at the solid/solution interface during the process of the adsorption of chlorpyrifos on ACCE/G (Table 3).

### 3.6 Regeneration and reuse of ACCE/G

**Fig. 6:**
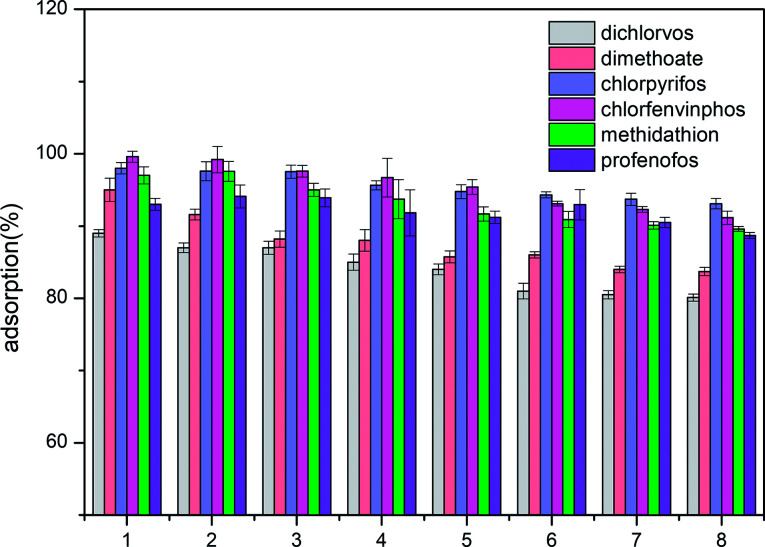
Adsorption efficiency of organophosphorus pesticides by ACCE/G after various cycles of regeneration.

Desorption experiments for the ACCE/G have also been carried out. Ethyl acetate has been chosen as the desorption solvent. Since the percentage of desorption is equal or higher than 90%, the ACCE/G could be recycled. The results of eight consecutive adsorption/desorption cycles are shown in Fig. 6. The adsorption efficiency of the ACCE/G for OPPs is still over 80% after the eighth cycle, proving that ACCE/G is a stable and efficient adsorbent for OPPs.

## 4. Conclusions

The AC derived from sieve-like cellulose–graphene oxide composite has an excellent adsorption capacity towards OPPs. Indeed, our newly synthesised composite material performs better than other five common adsorbents (multi-wall carbon nanotube, GCB, AC, C18, and PSA) in the removal of OPPs from water. The adsorption mechanism is mostly dependent on the electron-donating abilities of the S and P atoms. Moreover, the adsorption efficiency of the ACCE/G is still over 80% after eight times of recycling. Therefore, this work provides new insight into the application of the AC derived from corn straw as an adsorbent of pesticides in water.

## Conflicts of interest

There are no conflicts to declare.

## Supplementary Material

RA-008-C7RA12898C-s001
