# Supplementary material for: A carbonised sieve-like corn straw cellulose–graphene oxide composite for organophosphorus pesticide removal
Source: RSC Adv. 2018 Feb 16;8(14):7735–43. doi: 10.1039/c7ra12898c (PMC9078477; doi:10.1039/c7ra12898c)
Supplement: RA-008-C7RA12898C-s001 [file RA-008-C7RA12898C-s001.pdf]

Electronic Supplementary Material (ESI) for RSC Advances.
This journal is © The Royal Society of Chemistry 2018

Carbonised sieve-like corn straw cellulose-graphene oxide composite for organophosphorus pesticides removal

Fengyue Suo[a], Guixian Xie[b], Jie Zhang[c], Jingyu Li[c], Changsheng Li[c], Xue Liu[c], Yunpeng Zhang[c], Yongqiang Ma[c], MingShan Ji[a]

[a] College of Plant Protection, Shengyang Aguricultural University, Shenyang 110866, China

[b] College of Resources and Environment, Hunan Agricultural University, Changsha 410128, China

[c] College of Science, China Agricultural University, Beijing 100193, China

*Corresponding authors: MingShan Ji

Tel.: +86-024-88487148

Fax: +86-024-88487148

E-mail: mingshanji@163.com

School of Shengyang Aguricultural University

120 Dongling Road, Shengyang 110866, People's Republic of China

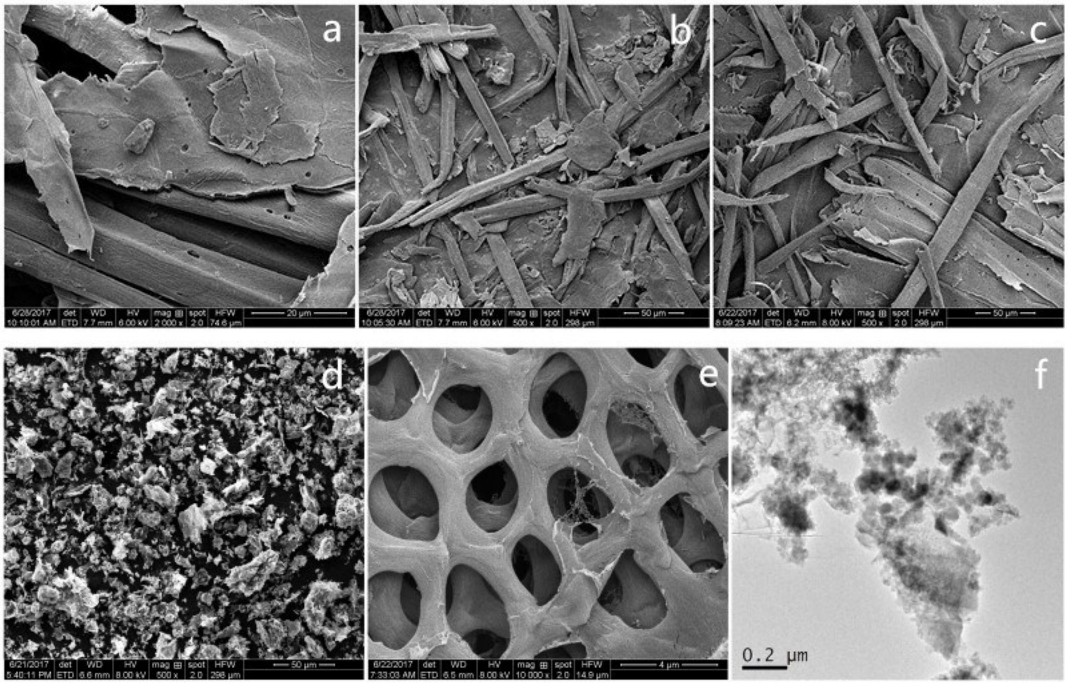

Fig. S1 the SEM images of cellulose (a)(b); the SEM image of CCE/G (c); the SEM image of GO (d); the SEM image of CCE (e); the TEM image of ACCE/G (f)

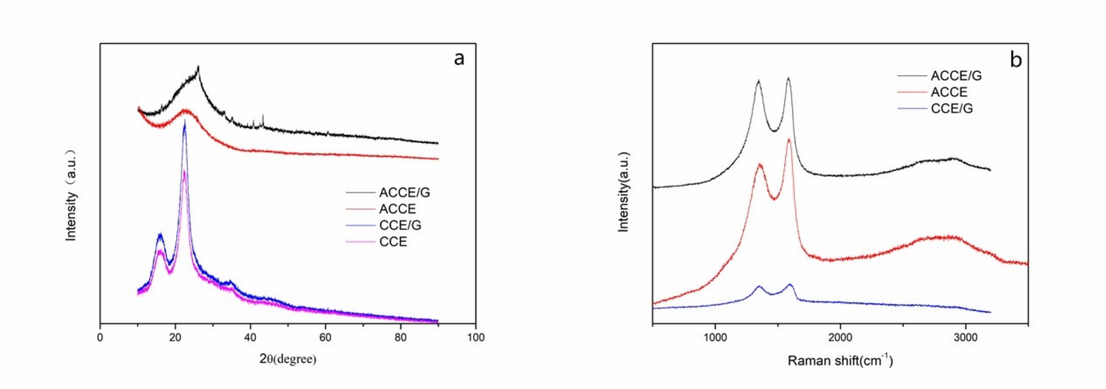

Fig. S2 X-ray diffraction patterns for CCE, CCE/G , ACCE and ACCE/G(a); Raman spectras of CCE/G, ACCE and ACCE/G (b)

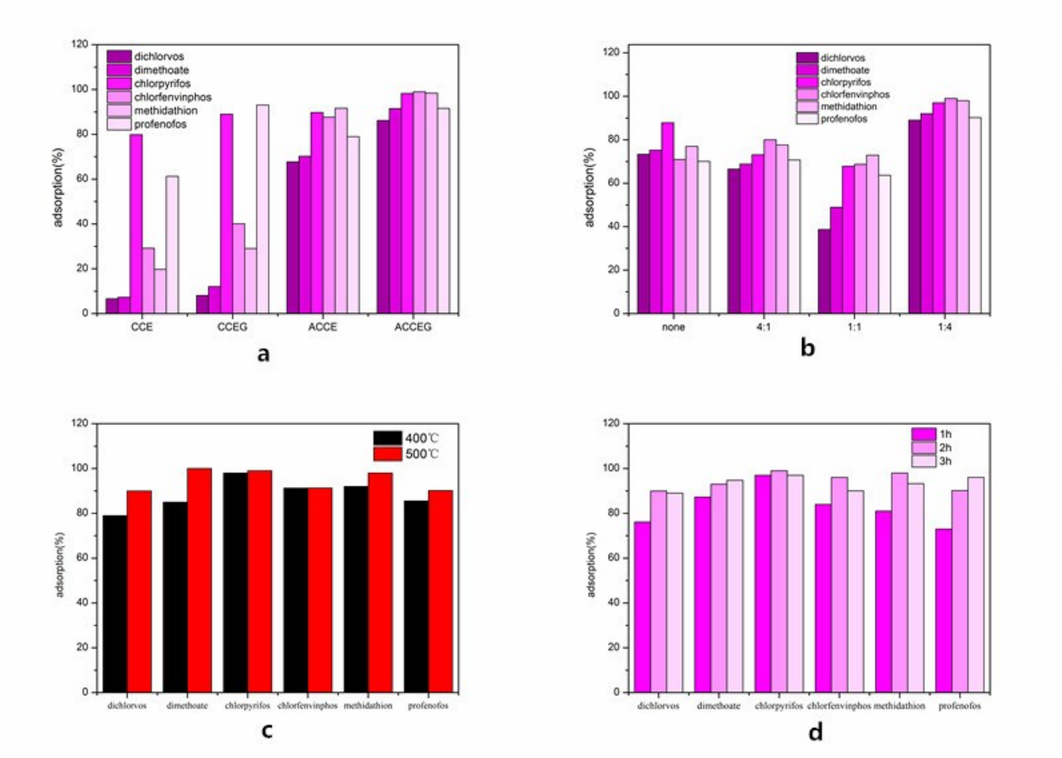

Fig.S3 Effects of different condition of synthesis on the adsorption efficiency: Heat-activated and have GO or not (a); The component of KOH(b); Heat-activated temperature(c); Heat-activated time(d).

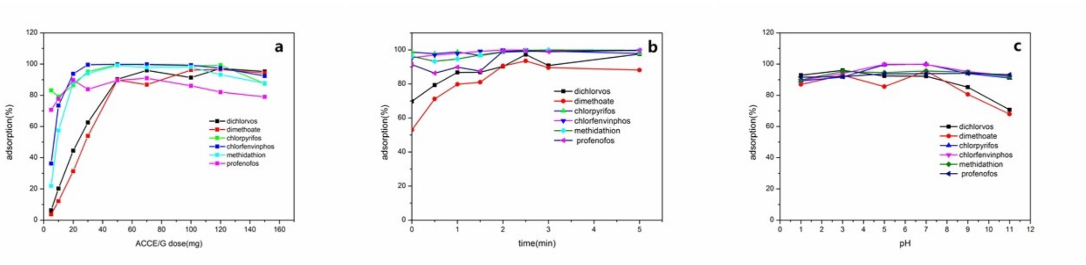

Fig.S4 Effect of the ACCE/G dose on the adsorption (a); Effect of the vortex time on the adsorption (b); Effect of pH on the adsorption (c)

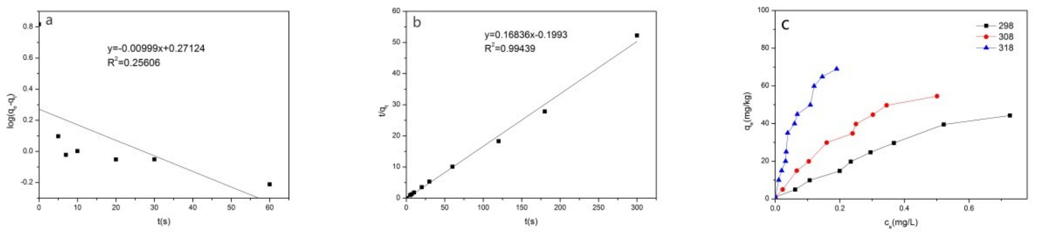

Fig.S5 Kinetic studies of chlopyrifos on to ACCE/G: Pseudo-first-order kinetics model (a); Pseudo-second-order kinetic model (b); Adsorption isotherms of chlopyrifos on ACCE/G (c)
